# Innovative Ultrasound-Assisted Approaches towards Reduction of Heavy Metals and Iodine in Macroalgal Biomass

**DOI:** 10.3390/foods10030649

**Published:** 2021-03-19

**Authors:** Estefanía Noriega-Fernández, Izumi Sone, Leire Astráin-Redín, Leena Prabhu, Morten Sivertsvik, Ignacio Álvarez, Guillermo Cebrián

**Affiliations:** 1 Department of Processing Technology, Nofima, NO-4021 Stavanger, Norway; Izumi.Sone@Nofima.no (I.S.); leena.prabhu@nofima.no (L.P.); Morten.Sivertsvik@Nofima.no (M.S.); 2 European Food Safety Authority, Via Carlo Magno 1A, 43126 Parma, Italy; 3 Departamento de Producción Animal y Ciencia de los Alimentos, Facultad de Veterinaria, Instituto Agroalimentario de Aragón—IA2—(Universidad de Zaragoza-CITA), 50013 Zaragoza, Spain; astrain@unizar.es (L.A.-R.); ialvalan@unizar.es (I.Á.); guiceb@unizar.es (G.C.)

**Keywords:** arsenic, cadmium, iodine, EDTA, brown macroalgae, *Laminaria hyperborea*, nonthermal technologies, mild heat, hurdle technology

## Abstract

The aim of this work was to evaluate the potential of ultrasound (US), alone or in combination with mild heating and/or EDTA towards reduction of As, Cd, I, and Hg content of *Laminaria hyperborea.* Concentrations of As, Cd, I, and Hg of 56.29, 0.596, 7340, and <0.01 mg kg^−1^ of dry weight, respectively, were found in *L. hyperborea* blades. Treatment with US at 50 °C increased approx. 2-fold the amount of As released, although did not affect significantly the content of Cd or I, as compared to control (no US) samples. Reducing the temperature to 8 °C significantly decreased the effect of US, but heating at 80 °C did not cause a significant effect as compared to treatments at 50 °C. On the other hand, treatment with 0.1 N EDTA at 50 °C enhanced the percentage of Cd released by approximately 7-fold, regardless of sonication. In the present work, the combination of US and EDTA at 50 °C for 5 min led to a significant reduction of the As (32%), Cd (52%) and I (31%) content in *L. hyperborea*, thus improving the product’s safety for consumers.

## 1. Introduction

Seaweeds have constituted a very relevant and recognized part of the diet in certain parts of the world, such as Japan, China and Korea, for centuries or even millennia [1]. Their use as food ingredients has recently gained attention in Western countries such as Europe and the US [2], which has been attributed to the rising popularity of Asian cuisine and the Western-consumers’ perception of seaweeds as a “healthy superfood” [3,4]. Nowadays, the annual production of algae for direct (sea vegetables) or indirect (phycocolloids) human consumption is estimated to be around 2,000,000 tons (dry matter) [5] with brown macroalgae being the most consumed macroalgae species (66.5%), followed by red (33%) and green (5%) seaweeds [6].

Seaweeds are rich in vitamins, minerals and essential trace elements, polyunsaturated fatty acids, bioactive metabolites, proteins, polysaccharides, and dietary fiber [7]. Furthermore, health-promoting effects have been associated to direct seaweed consumption and the use of their extracts/components as processing aids (e.g., as hydrocolloids, emulsifiers and/or gelling agents) and dietary supplements [5,7,8,9,10,11]. However, their substantial bio-sorption and bio-accumulation capacity [12], particularly that of brown algae species such as *Laminaria hyperborea*, may increase the dietary exposure to potentially harmful compounds. Among them, heavy metals such as inorganic arsenic (iAs) and cadmium (Cd), and the essential micronutrient iodine (I), whose excessive intake may lead to adverse health effects [13], have been reported as major chemical hazards associated to seaweed consumption in the European seaweed chain [3]. Thus, several studies have reported high levels of iAs, Cd and I in seaweed and seaweed-based food products, which in many cases exceeded the recommended or allowed levels in different countries [14,15,16,17,18,19,20]. Nevertheless, it also should be noted that levels of heavy metals and iodine in seaweeds have been reported to substantially vary across species, location, season, and growth-related factors [21,22,23,24].

Different post-harvest processing strategies have been assessed to reduce the content of undesirable elements in seaweeds. Traditional methods include rinsing, boiling, fermentation and drying. Rinsing in water has been shown to reduce iAs and/or I levels [15,23,25,26,27], although no significant effects have otherwise been reported in a few studies [28]. Temperature seems to play a key role as reductions in I and As up to 94% have been observed after blanching or boiling [17,25,29]. For instance, the Belgian Superior Health Council has recommended boiling as an effective method for iAs removal in seaweeds, although the relatively high levels of As released into the water may limit further uses of the cooking media [30]. Similarly, fermentation has also been demonstrated as an effective strategy to reduce the content of Hg, Cd and I in sugar kelp [31]. However, the efficacy of these methods seems to vary widely with operational conditions, seaweeds species, etc., leading most of them to undesirable changes in the nutritional and organoleptic properties of seaweeds [32,33,34,35,36].

In the last decade, promising potential of innovative nonthermal technologies, such as ultrasound (US), has been demonstrated for green extraction of algal bioactives (e.g., carotenoids, polyphenols, polysaccharides), with improved process cost-effectiveness and recovery yield, and enhanced techno-functionality and bioactivity of end products [37]. US, typically combined with mild heating, acid or alkali surfactants, has also been proved as an effective preparative step for improved extraction of heavy metals and iodine from seaweeds towards further quantification via e.g., Inductively Coupled Plasma Optical Emission Spectrometry (ICP-OES) or Inductively Coupled Plasma Mass Spectrometry (ICP-MS) [38,39,40,41,42]. However, apart from these applications that require high US intensities in order to disrupt seaweed cells, this technology can also be used to enhance mass and energy transfer processes when applied at much lower intensities. This would limit its impact in the organoleptic properties of seaweeds, enabling the production of seaweed-based products with enhanced quality attributes (e.g., texture, color, flavor) while ensuring food safety and/or extended shelf-life, and would also facilitate its industrial implementation because of the lower associated costs [43,44,45]. However, to the knowledge of the authors, the potential of US to reduce the content of heavy metals and I in food matrices, and particularly in seaweeds, has been hardly investigated. Very promising results were reported by Condón-Abanto et al. [46], who demonstrated that the combination of US and mild heating effectively reduced the content of Cd in edible crab but, still, further studies focusing on process characterization and optimization on relevant seaweed matrices, as well as synergistic and multitarget combination with relevant hurdles (e.g., temperature, EDTA), are required towards technology validation and eventually, industry uptake.

Therefore, the aim of this work was to evaluate the potential of US, alone or in combination with mild heating and/or EDTA, a food-grade chelating agent with potential to desorb metal ions from macroalgae [47], to reduce the content of iAs, Cd, Hg, and I in wild-harvested *L. hyperborea.*

## 2. Materials and Methods

### 2.1. Raw Material

Wild *Laminaria hyperborea* samples were kindly supplied by Dolmøy House of Seafood AS (Frøya, Norway). Whole mature plants, harvested in May 2020 at 63°41′49.2′′ N 8°49′22.7′′ E, were shipped overnight under refrigerated conditions. Immediately after reception, both stipe and holdfast of selected plants were removed with a sterile scalpel and discarded. The 2–3 cm-side square-shaped samples were aseptically cut from central undamaged areas of the remaining blades, then placed into sterile glass jars closed with aluminum lids (5.0 ± 0.2 g total wet weight per jar) and stored at 4 °C overnight.

### 2.2. Treatment Conditions

Glass jars with ≈5 g *L. hyperborea* samples were filled in with 100 mL Milli-Q water tempered as appropriate, and treated at 8, 50 or 80 °C for 5 and 30 min either in a thermostatic water bath (Anritsu HD-1250K Thermometer, Atsugi, Japan) or an ultrasonic water bath operating at 68 kHz and 500 W (BT 130H, UPCORP, Freeport, IL, USA), with a nominal specific power of 0.016 W/g. Likewise, 5 g macroalgae samples were exposed to 100 mL 0.1 N EDTA disodium salt (Sigma-Aldrich, Darmstadt, Germany) for 5 and 30 min at 50 °C, both in presence and absence of sonication. Under the assayed conditions, the final temperature of the treatment media increased less than 5 °C as a consequence of the application of ultrasound (data not shown).

After the treatment, seaweed samples were recovered from the rinsing media and then dehydrated at 105 °C in a convection drying oven (P-model Digitronic Selecta, Barcelona, Spain). After weighing, dehydrated samples were ground and stored in hermetic tubes until further analysis of iodine and heavy metals.

The content of heavy metals (arsenic, cadmium and mercury) and iodine was also analyzed in the corresponding rinsing media. HNO_3_ (J.T. Baker, Philipsburg, New Jersey, USA) or TMAH (25% *w*/*w*, Sigma-Aldrich) were added, respectively, to those samples intended for analysis of heavy metals and iodine, at a final concentration of 5% *v*/*v*. The samples were then frozen at −30 °C until further analysis.

### 2.3. Cadmium, Mercury and Total Arsenic Analysis

The amount of cadmium, mercury and total arsenic in both seaweed and rinsing media samples was measured by ICP-MS. For seaweed samples, 0.2 g of dehydrated seaweed was diluted in MilliQ-water and incubated at room temperature for 20 min in microwave-resistant tubes (MarsX-press Plus Wessel, CEM Corporation). Then, 5 mL HNO_3_ was added to the tubes, which were sealed right after. Samples were digested in a microwave system using a 10-min heating ramp at 800 W and maintained at 200 °C for 10 min. Then, the samples were cooled, filtered (0.45 µm) and adequately diluted prior to injection in the ICP-MS equipment (Elan DRC-e, Perkin-Elmer, Rodgau, Germany). The ICP-MS instrumental settings are described in Condón-Abanto et al. [46]. Samples of treatment media were processed in a similar way, but 5.0 mL of sample, instead of 0.2 g of dehydrated sample, was directly mixed with 5 mL HNO_3_ within the microwave tubes before the digestion step.

### 2.4. Iodine Analysis

The amount of iodine was measured by means of ICP-OES. For seaweed samples, 0.2 g of dehydrated seaweed was diluted in 5 mL MilliQ water within gas-tight glass tubes (Sovirell tube). TMAH (25% *w*/*w*, Sigma-Aldrich) was added to a final concentration of 5% *v*/*v* and tubes were closed with screw caps and PTFE-coated rubber sealing. Samples were digested in a convective oven at 90 °C for 3 h. Then, samples were cooled, filtered (0.45 µm) and adequately diluted before being injected in the ICP-OES equipment (Elemental IRIS Intrepid II XLD, Thermo Electron, Waltham, MA, USA). Samples of treatment media were processed similarly, but 5.0 mL of the treatment medium, instead of 0.2 g of dehydrated sample, was added to the tube before the addition of TMAH and the digestion step.

### 2.5. Determination of Removal Efficacy

The results obtained from the seaweed analysis were expressed as mg of As, Cd, Hg, and I per kg of dry weight (ppm). The amount of each compound released was calculated using Equation (1):

(1) $$\%\;substance\;released=\frac{{\left[substance\right]}_{water}\;\times \;V\;\times \;100}{{\left[substance\right]}_{seaweed}\;\times \;W}$$

where *[substance]_water_* represents the concentration of the substance in mg/L measured in the treatment medium; *V*, is the volume of treatment medium (0.1 L); *[substance]_seaweed_* is the concentration of the substance in mg/kg dry weight in the fresh (untreated) seaweed and *W* is the dry weight (kg) of the fresh seaweed sample.

### 2.6. Statistical Analysis

GraphPad PRISM software (Graph Software, San Diego, CA, USA) was used for statistical analyses (analysis of variance and Student’s *t*-test) (*p* = 0.05). All the determinations were performed in triplicate.

## 3. Results

### 3.1. As, Cd, Hg, and I Content in Untreated, Wild-Harvested L. hyperborea

The content of As (total), Cd, Hg and I was determined in untreated, wild-harvested *L. hyperborea* samples in order to set a biological baseline for further estimations of the removal efficacy of US alone or in combination with mild heating and EDTA. Despite the content of Hg being below the IPC-MS detection limit, the levels determined for the remaining compounds fall within the concentration ranges reported in the literature for *L. hyperborea* and other brown macroalgae [16,23,48,49,50,51]. Variability in the content of As, Cd, Hg, and I in *L. hyperborea* and, in general, seaweeds, has been substantially reported. Geographical location, seasonality, salinity, and temperature of the surrounding water, depth, and developmental determinants, such as maturity and blade morphology and size, among others, account for such variability [23,48,51,52,53,54,55]. Thus, in preliminary experiments, up to 56% variability in the content of Cd (data not shown) was found in *L. hyperborea* samples collected within a one-month span across the harvesting season. Considering such a high fluctuation in the raw material, the present work was conducted with mature *L. hyperborea* specimens with similar morphological features (i.e., blade size and width), which were harvested simultaneously from the same location. Nonetheless, the biological variability (standard deviation of three replicates) in the content of heavy metals and iodine in the raw samples accounted for more than 10% of the mean values for all the elements studied, and up to 23.7% for the I content, which was attributed to the significant variability across specimens. To avoid interferences in the analytical determinations derived from such a high biological variability in the raw material, the amount of heavy metals and iodine released into the rinsing media was measured rather than the remaining levels in the blades after the respective treatments, as already described elsewhere [46].

In the present study, total As levels in *L. hyperborea* blades (56.3 ± 5.1 mg/kg), similar to those reported for *L. digitata* (36.0–131.0 mg/kg of dry weight), were however higher than the values found in other macroalgae species [16,56,57,58] but lower (5-fold) than those reported for Hijiki [29]. Arsenic is a metalloid present in the environment in different inorganic (iAs) and organic forms, originating from natural and anthropogenic sources [59]. Due to its high concentration (more than 10-fold higher than the content in vegetables, grains and meats), seaweed intake greatly contributes to the dietary exposure to As [18,26,60]. For instance, the Korea Food and Drug Administration reported that seaweed accounted for the second highest source of exposure to total arsenic via food (20%) in Korea, despite seaweeds ranking 13th out of 17 items in the daily per capita intake, with 22.4 g/person/day [29]. iAS forms (arsenate V (Asv) and arsenite III (AsIII)) are acknowledged to be more toxic than organic forms [61] and, although in the majority of species most of the arsenic exists in the form of nontoxic arsenosugars, iAs can account for up to 60–73% of the total As for some of them [60,62]. Assuming that only 10% of the total As in the samples would be in inorganic form (up to 50% has been reported for this particular species [57]), the As content reported in the present work would exceed the limit of 3 mg/kg dw iAs recommended by the French Food Safety Authority in edible seaweeds [63,64].

The content of Cd in untreated *L. hyperborea* samples determined in the present study (0.6 ± 0.01 mg/kg) is lower than the levels reported for *L. japonica* (0.45–0.80 mg/kg) [26], but still well-above the maximum limits set for seaweed products by the Food Safety Authority in France (0.5 mg/kg of dry weight) and Australia/New Zealand (0.2 mg/kg of dry weight) [65]. However, these concentrations would be reasonably close to the maximum permitted levels set by the Food Safety Authority in China (1.0 mg/kg) [65] and in Regulation (EC) 1881/2006 (3.0 mg/kg of wet weight) for food supplements consisting exclusively or mainly of dried seaweed, products derived from seaweed, or of dried bivalve molluscs [3].

Although the content of Hg in *L. hyperborea* was below the detection limit (<0.01 mg/kg) of the analytical technique (ICP-MS), and thus excluded for further analysis, the concentration range coincides with values reported for brown seaweeds [16]. Moreover, the ICP-MS method’s performance criteria would still fall far below the maximum permitted level (0.1 mg/kg) set by the Regulation (EC) 1881/2006 in food supplements and by the French Food Safety Authority in edible seaweeds [3].

Finally, the I values determined in this work (7340 ± 1744 mg/kg) largely exceed the maximum limit recommended by the French Food Safety Authority (2000 mg/kg dw) in edible seaweeds [66]. The substantial accumulation of this micronutrient in brown macroalgae and particularly *Laminariales* (>30,000-fold higher than environmental levels) has been attributed to the high content of alginate and sulphated polysaccharides and the presence of cell-wall haloperoxidases [16,48,51]. Despite the benefits for iodine-deficient populations, highly susceptible to goiter and hypothyroidism, excessive intake of iodine can however cause adverse health effects such as dysfunctions of the thyroid gland [67,68]. Thus, dietary reference values for I, expressed as adequate intake (AI), have been set as 150 μg/day for adults, 70–100 μg/day for infants and children, and 200 μg/day for pregnant and lactating women [68]. At the same time, tolerable upper intake levels of 600 μg/day for adults, including pregnant and lactating women, and 200–500 μg/day for children, have been recommended. However, the average dietary iodine intake due to the ingestion of seaweeds has been estimated for instance in Japan as 1–3 mg/day, which significantly exceeds tolerable upper levels [69].

In conclusion, the relatively high content of As, Cd and I in brown macroalgae, as confirmed in the present work, seems to exceed maximum permitted levels and substantially contribute to the dietary intake of such potentially harmful compounds, which is urging the implementation of sustainable and multitarget removal strategies towards improved food safety of seaweeds and products thereof.

### 3.2. Effect of US on As, Cd and I Removal from L. hyperborea Blades

**Figure 1:**
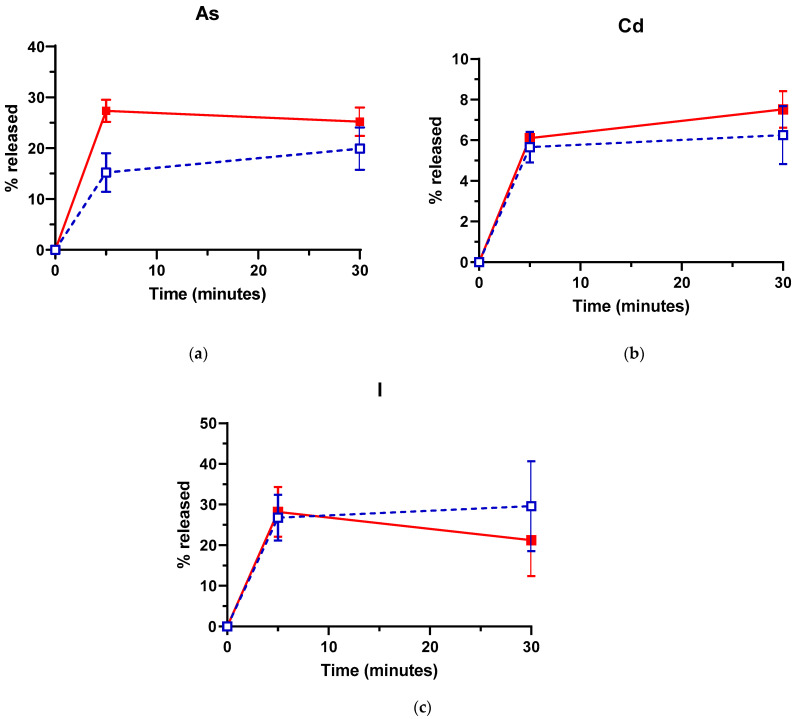
Percentage of arsenic (As) (**a**), cadmium (Cd) (**b**) and iodine (I) (**c**) released from *Laminaria hyperborea* blades after Ultrasound (US) (red filled symbols, continuous lines) and Control treatments (no US; blue empty symbols, discontinuous lines). Treatment temperature: 50 °C. Error bars correspond to the standard deviations.

The potential of US for increasing and/or accelerating the release of As, Cd and I from *L. hyperborea* blades was initially studied at 50 °C, which has been reported as the optimal temperature for US-assisted removal of Cd in crab claws [46]. Moreover, this value is very close to the minimum temperature (45 °C) necessary for significantly reducing the iodine content of *Sacharrisima lattisima* after soaking treatments [25]. As observed in Figure 1a, US treatment significantly increased the amount of As released into the medium from *L. hyperborea* blades, almost doubling after 5 min the levels released in absence of US (27.4% vs. 15.2%). Longer US treatment time (30 min) did not significantly improve the removal efficacy for As, although a slight increase in average values (not statistically significant) was observed for control samples in absence of US. However, the amount of As released after 30 min at 50 °C was still significantly higher for US-treated samples. Regarding Cd and I, no significant differences in the removal efficacy were observed for sonicated and control samples, independently of the treatment time (Figure 1b,c).

These results might be explained on the basis of, on one hand, the different mechanisms of bio-absorption and bio-accumulation in macroalgal biomass and, on the other hand, the mechanism of action of US. Thus, chelation is the main sequestration mechanism for Cd, whereas adsorption and metabolism-regulated active uptake have been reported for As [70,71]. Interestingly, As might be stored in inorganic form (>50% for some species) or most commonly as nontoxic arsenosugars, which depends on the particular macroalgae species. Moreover, interspecies differences in cell-wall composition and chemistry seem to play a major role in the biphasic accumulation of heavy metals (e.g., As vs. Cd), which is regulated first by rapid extracellular adsorption or passive intracellular uptake, and thereafter by metabolism-dependent incorporation and excretion processes. For instance, higher affinity for heavy metals has been reported in macroalgal biomass rich in carboxyl groups rather than sulphate groups in the cell wall [72]. These compositional differences across species might lead in some cases to most of the As and Cd (60-80%) being retained at the apoplast or surface [73]. Regarding iodine, 99% content in *Laminariales* has been reported to be highly water soluble [74] since the majority would be occurring as noncovalently bound I− in the apoplast outside the cells [75].

Considering the low US intensity applied, breakdown of seaweed cell walls/membranes or covalent bounds is not expected, as structural modifications of the blades were not observed after the US treatments at 50 °C (data not shown). Moreover, the potential contribution of US-generated free radicals to the release of As, Cd and I is expected to be negligible due to the low US intensity and frequency (68 kHz) applied. Therefore, US treatment would only enhance the release of those elements located in the apoplast or surface of the blades and not covalently bound to other molecules (unless the whole molecule is released to the medium). Noncovalently bound I and inorganic As would then leach rapidly to the treatment medium (distilled water), whereas chelated Cd will not be released to the same extent. The present study supports the above-mentioned assumptions, with 30% removal efficacy for I and As after US treatment, as compared to 9% for Cd. In addition, US might not have a significant effect on I release, as compared to the soaking/washing treatment itself (with no US), given its high solubility in water. As the results obtained for As suggest, US treatment most likely will not lead to higher decontamination level but otherwise accelerate desorption kinetics. Therefore, a more pronounced effect of US on the amount of I released, as compared to control samples, might have been observed at shorter treatment times. Finally, the apparent inefficacy of US towards Cd and I release, as compared to control samples, might be attributed as well to the low US intensity applied in this work, which leaves room for improved US decontamination efficacy in further works.

The outcomes of the present study differ from the results presented by Condón-Abanto et al. [46], where a linear increase in the removal efficacy for Cd was reported in crab claws towards increasing US treatment time, eventually achieving 9-fold higher values than those in control samples. These differences may be attributed to different US operational conditions (e.g., frequency and mode of application) and treatment media (tap vs. distilled water), as well to the different Cd bioaccumulation mechanisms in seaweeds and crustaceans and to the differences between animal and seaweed cell structure/envelopes. Further studies are required in order to shed light on the release mechanisms for As and Cd, as well as to determine the specific effect of US on the removal of inorganic As.

### 3.3. Influence of Treatment Temperature on As, Cd and I Removal by US

**Figure 2:**
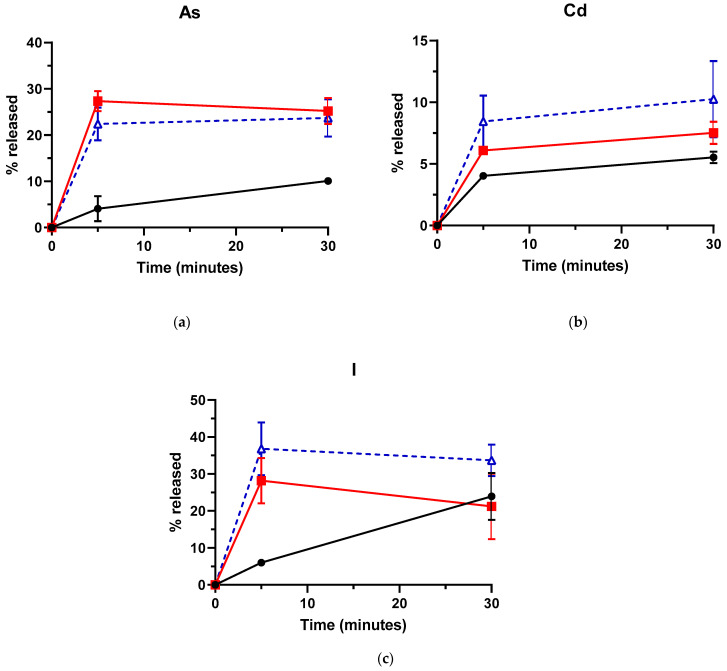
Influence of treatment temperature on the percentage of arsenic (As) (**a**), cadmium (Cd) (**b**) and iodine (I) (**c**) released from *Laminaria hyperborea* blades after Ultrasound treatments. Treatment temperature: 8 °C (●), 50 °C (■) and 80 °C (**△**). Error bars correspond to the standard deviations.

Treatment temperature has been demonstrated to play a major role in the release of undesirable compounds from seaweeds [15] and to enhance US food preservation efficacy [76]. Thus, the influence of treatment temperature on As, Cd and I release after US treatments was studied at 8, 50 and 80 °C for 5 and 30 min. As observed in Figure 2, a temperature rise from 8 to 50 °C resulted in a significant (*p* < 0.05) increase in the removal of As and Cd for both 5 and 30 min US treatments, whereas the longest treatment time was needed to enhance significantly the I released. However, no significant differences in As, Cd and I release were found when US treatments were carried out at 50 or 80 °C, regardless of the treatment time, which might be attributed to (1) the reduced energy transferred by cavitation when the vapor tension of the liquid increases [77,78], and (2) the limited effect of US on Cd and I release. It is noteworthy that, in absence of US treatment, the temperature increase from 50 to 80 °C did not improve either the release of these compounds from *L. hyperborea* blades (data not shown). Although Condón-Abanto et al. [46] also reported 50 °C as the optimal temperature for US removal of Cd in edible crab, the high biological variability across macroalgal specimens might have dampened the actual effect of the treatment temperature, at least for Cd and I. On the other hand, US treatment time (5 or 30 min) only played a significant role at 8 °C, with regards to the amount of As and I released. Therefore, US treatments as short as 5 min would lead to the maximum decontamination efficacy, provided that the temperature is ≥50 °C, which would save time and resources and thus, facilitate the industrial implementation of this technology.

### 3.4. Influence of the Addition of EDTA on As, Cd and I Removal by US

**Figure 3:**
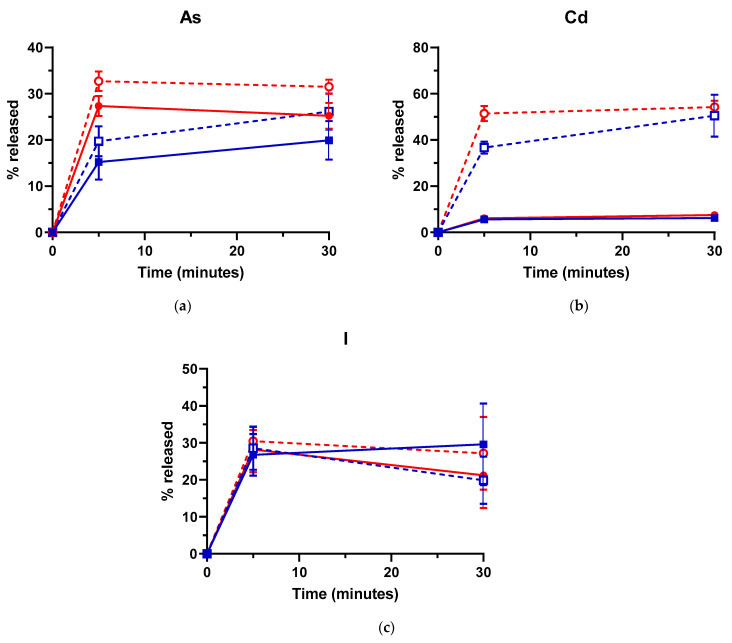
Percentage of arsenic (As) (**a**), cadmium (Cd) (**b**) and iodine (I) (**c**) released from *Laminaria hyperborea* blades after Ultrasound (US) (red symbols and lines: ●, ○) and Control treatments (no US; blue symbols and lines: ■, ☐). Treatments were carried out with (empty symbols, discontinuous lines: ○ - -, ☐ - -) or without (filled symbols, continuous lines: ●, ■) the addition of EDTA (0.1 N). Treatment temperature: 50 °C. Error bars correspond to the standard deviations.

The potential of US treatment in combination with EDTA (0.1 N) for the removal of As, Cd and I is shown in Figure 3. Since (1) the removal efficacy for As and Cd significantly decreased at 8 °C and (2) no significant differences were found in the % released at 50 and 80 °C, the former temperature was selected for these trials. The addition of EDTA resulted in a significant increase (between 6.5- and 8.4-fold) in the amount of Cd released, regardless of sonication and treatment time. This increase was even slightly higher for US treatments. Thus, although no significant differences in the amount of Cd released were found between control and US-treated samples in absence of EDTA, they were found (*p* < 0.05) for 5 min treatments when this chelating agent was added to the treatment media (Figure 3b).

Although in a less pronounced manner, addition of EDTA also increased the amount of As released (1.2- and 1.3-fold; Figure 3a), although did not improve the removal efficacy for I, irrespective of sonication and treatment time (Figure 3c). EDTA is a strong chelating agent approved in the EU as a food additive (E385) and in the United States (GRAS status). Its high level of complexing capacity with regard to heavy metals [79] would explain the increased release of Cd from *L. hyperborea* blades, thus supporting chelation as the main macroalgae sequestration mechanism for the Cd^+2^ cation. On the other hand, the limited effect of EDTA on As could be attributed to the desorption of organic arsenic species not being affected by EDTA addition, since coprecipitation only occurs with inorganic species [80]. Strong chelating agents such as EDTA have been reported to break down cellulose polymers and degrade macroalgal cell wall [12,29], thus leading to complete solubilization of the biomass and release of both anionic and cationic species. However, no biomass loss was observed upon the EDTA addition in this study, which would explain why the release of I was not affected by EDTA addition.

## 4. Conclusions

Accumulation of As, Cd and I in brown macroalgae stands as a major food safety hazard restraining the broad utilization of this valuable nutrient source in food applications [15,81]. The relatively high content of As, Cd and I in brown macroalgae, as confirmed in the present work, seems to exceed maximum permitted levels and substantially contribute to the dietary intake of such potentially harmful compounds, which is urging the implementation of sustainable and multitarget removal strategies towards improved food safety of seaweeds and products thereof. In the present study, the combined application of US, mild heating (50 °C) and EDTA (0.1 N) for 5 min led to 32% and 52% release of As and Cd, respectively, from *L. hyperborea* blades without affecting the efficacy of soaking treatments (at the same temperature) towards reduction of I content. To the knowledge of the authors, this is a pioneering study demonstrating the potential of combined US, EDTA and mild heat for reducing the As, Cd and I content in seaweed. However, further work on process optimization, with particular emphasis on US intensity and frequency and the rinsing media, as well as interspecies validation is required towards both maximum removal of As, Cd and I and minimum impact on nutritional and sensory quality of kelp.

## Data Availability

The data presented in this study are available on request from the corresponding author.

## Disclaimer

The author Estefanía Noriega Fernández, is employed with the European Food Safety Authority (EFSA) in the Unit Nutrition that provides scientific and administrative support to the Panel on Nutrition, Novel Foods and Food Allergens in the area “Safety Assessment of Novel Foods”. However, the present article is published under the sole responsibility of the authors E. Noriega Fernández, I. Sone, L. Astráin-Redín, L. Prabhu, M. Sivertsvik, I. Álvarez, G. Cebrián and may not be considered as an EFSA scientific output. The positions and opinions presented in this article are those of the author/s alone and are not intended to represent the views/any official position or scientific works of EFSA. To know about the views or scientific outputs of EFSA, please consult its website under http://efsa.europa.eu.
